# Early Postnatal B Cell Ontogeny and Antibody Repertoire Maturation in the Opossum, *Monodelphis domestica*

**DOI:** 10.1371/journal.pone.0045931

**Published:** 2012-09-24

**Authors:** Xinxin Wang, Alana R. Sharp, Robert D. Miller

**Affiliations:** Center for Evolutionary and Theoretical Immunology, Department of Biology, University of New Mexico, Albuquerque, New Mexico, United States of America; INRA, France

## Abstract

Marsupials are a lineage of mammals noted for giving birth to highly altricial young, which complete much of their “fetal” development externally attached to a teat. Postnatal B cell ontogeny and diversity was investigated in a model marsupial species, the gray short-tailed opossum, *Monodelphis domestica.* The results support the initiation of B cell development late in gestation and progressing into the first two weeks of postnatal life. Transcription of CD79a and CD79b was detected in embryonic tissue prior to birth, while immunoglobulin heavy chain locus transcription was not detected until the first postnatal 24 hours. Transcription of the Ig light chains was not detected until postnatal day 7 at the earliest. The predicted timing of the earliest appearance of mature B cells and completion of gene rearrangements is consistent with previous analyses on the timing of endogenous antibody responses in newborn marsupials. The diversity of early B cell IgH chains is limited, as has been seen in fetal humans and mice, but lacks bias in the gene segments used to encode the variable domains. Newborn light chain diversity is, from the start, comparable to that of the adult, consistent with an earlier hypothesis that light chains contribute extensively to antibody diversity in this species.

## Introduction

The degree of immunological competence of newborn animals varies considerably between mammalian species. A newborn mouse, for example, is much less developed than the more immunologically precocious cow or pig [1], [2]. Whether a species is considered altricial or precocial at birth is, of course, a relative distinction [3]. The marsupials are one of three living lineages of mammals (placentals, marsupials, and monotremes [*e.g.* the egg laying platypus]) that differ substantially in their state of development at birth. Marsupials, such as opossums and kangaroos, are born in an extreme altricial state compared to any placental mammal. The developmental state of the newborn marsupial immune system has been equated to that of a human embryo at the eighth to tenth week of gestation or a mouse or rat at the tenth day of gestation [4]–[6]. Therefore, much of the development that occurrs in prenatal humans and other placental mammals appears to be postnatal in marsupials, making marsupials unique models of early immune system development.

Indicative of their altricial state, newborn marsupials are unable to initiate endogenous immune responses until they are at least a week of age [6]. The North American opossum *Didelphis virginiana*, for example is unable to generate a T-dependent antibody response until greater than seven days of age [4], [7], [8]. Similar results have been found with other marsupial species [9]. The ability to generate cell-mediated immune responses such as allograft rejection also develops postnatally in marsupials. Skin grafts are tolerated for most of the first two postnatal weeks of age in opossums and macropods [10], [11]. However, allografts are rejected at later ages consistent with the eventual appearance of functional T cells. The postnatal development of immune-competence is also consistent with the appearance of cells expressing lymphocyte markers in newborn marsupials [6]. In tammar wallabies, for example, lymphocytes expressing the B cell marker CD79b (Ig-β) could be found in the gut associated lymphoid tissue of pouch young as early as day 7 [12]. Whereas CD3^+^ lymphocytes were not detected in the thymus until postnatal day 12.

Placental mammals can differ in the diversity of the antibody repertoire generated during fetal development when compared with that of the adult. One mechanism for this difference has been associated with the lack of non-templated (N) nucleotide additions in the junctions between the variable (V), diversity (D), and joining (J) gene segments during the recombination of the gene segments that encode the V domain of immunoglobulin heavy (IgH) chains. This is due to low or absent expression of the enzyme Terminal deoxynucleotidyl Transferse (TdT) in early developing B cells [13]. The absence of N-additions is thought to achieve multiple goals in early B cell development. For one, it is associated with bias in VDJ recombination driven by short sequence homology. Such bias appears to be one mechanism for preferentially generating beneficial idiotypes specific for common pathogens such as the protective anti-phosphorylcholine response in mice [14]. In addition, B cells expressing IgH lacking N-additions more rapidly populate lymphoid tissues, which may be advantageous early in ontogeny when first seeding peripheral sites [15]. This appears to be at the expense of more efficient antibody responses, however, presumably due to a more limited IgH chain repertoire. Limited N-additions in early B cell development is not universal to all placental species. In pigs for example there is limited diversity of IgH complementarity determining region-3 (CDR3) in early fetal development, but this is not due to lack of N-additions. Rather it is likely due to limited B cell numbers early in ontogeny [16]. Unlike humans and mice, pigs have restricted recombinatorial diversity, using a limited variety of V, D, and J segments to derive their IgH repertoire. Pigs may have compensated for this limitation through increased CDR3 diversity earlier in development than is found in humans and mice [16].

In species such as marsupials, where B cell competence appears to develop postpartum, it is not known whether there are changes in the repertoire that are analogous to the fetal-to-adult transition found in humans and mice. Here we investigate that question and established the timing of critical steps in B cell development in a model marsupial species.

The gray, short-tailed opossum, *Monodelphis domestica* is arguably one of the better-established marsupial species for biomedical research [17], [18]. They are easily bred in captivity, are not seasonal breeders, and are pouchless providing easy access to large litters of newborn opossums while they remain attached to the teats [18]. A high quality whole genome sequence is available and the content and organization of their germ-line T cell receptor (TCR) and Ig genes has been established [19]–[21]. The opossum has single IgM, IgG, IgE, and IgA isotypes, along with both the Igκ and Igλ L chains [21]–[25]. *M. domestica* lacks the genes for IgD [21]. The IgH locus contains three VH families that are all closely related within the ancient VH clan III [21], [22]. Family VH1 is composed of 24 V gene segments of which 5 are pseudogenes. Families VH2 and VH3 each contain a single, functional gene segment, however VH3 is atypical in that it is germ-line joined to a DH segment, and is the only known germ-line joined VH gene found in mammals. [21]. VH3.1 can be recombined directly to a JH segment and is transcribed although appears to be rarely used and was only detected in the IgH repertoire later in development [26]. In contrast to the IgH chains with limited germ-line VH diversity, the opossum Ig light chains have a diverse set of germ-line V genes [21], [27]. There are 122 V genes divided into seven families in the Igκ locus and 64 V gene segments divided into four families in the Igλ locus. The higher level of germline diversity in Ig light chain genes appears to be common across a broad spectrum of marsupials and has lead to speculation that light chains contribute more to antibody diversity than do heavy chains in this lineage [27].

Utilizing the available genomic information for Ig genes and B cell markers the ontogeny of the Ig repertoire and timing of B cell development was investigated in the opossum.

## Materials and Methods

### Ethics Statement

All procedures using live animals were conducted under the approved under the Institutional Animal Care and Use Committee of the University of New Mexico (Protocol number 07UNM005). No live surgery was performed.

### Tissue Collection, RNA Extraction and Complementary DNA (cDNA) synthesis

*M. domestica* typically give birth in the evening and, for the purposes of this study, neonates collected the next morning were counted as being postnatal day 1 (P1). Due to their small size, opossums less than P10 in age were either extracted whole or using the abdominal region containing the liver, gut, spleen, and bone marrow. For opossums P10 and older individual tissues were collected. For embryonic tissues, pregnancies were timed from the point of ovulation, which occurs on average five days following the pairing of females with a male [28].

All tissues were either used immediately or stored in RNAlater (Ambion, Austin, TX) at 4°C for 24 hours and long term at –80°C. Total RNA was extracted using Trizol RNA extraction protocol (Invitrogen, Carlsbad, CA). Reverse transcription-polymerase chain reaction (RT-PCR) was performed using the Superscript III First-Strand kit (Invitrogen, Carlsbad, CA).

### PCR and Sequencing

PCR amplification was performed using Advantage TM-HF 2 PCR (BD Biosciences, CLONTECH Laboratories, Palo Alto, California). PCR products are cloned using TOPO TA cloning Kit (Invitrogen, Carsbad, CA) and sequenced using BigDye Terminator Cycle Sequencing Kit (Applied Biosystems, Foster City, CA). All sequences reported are based on sequencing both strands of each clone. Chromatograms are analyzed using Sequencher 4.9 (Gene Codes, Ann Arbor, MI).

### Ig transcript Collection and Junctional Diversity Analyses

To amplify Ig transcripts, forward primers were designed individually complementary to each V gene family. Forward primers that are complementary to framework region (FR) -1 in the VH1, VH2 and VH3 families are: 5′-CCTGCAAAGCTTCTGGATTC, 5′-CATGCATTGGATACGACAGG and 5′-GGACATCTCTGCACCTCTCC, respectively. Forward primers complementary to FR1 of Vκ1, Vκ2, Vκ3, Vκ4, Vκ5, Vκ6 and Vκ7 families are: 5′-TCCCTGGCTGTGTCTC(T,C)AGG, 5′-CCAGCCTCTGTGTCTGTGTC, 5′-TGTGATGACCCAGACTCCAG, 5′-TCCAGCCTCTTTGTCCAGAT, 5′-TCCATCCTCTCTGTCTGCAA, 5′-AATCTCCTGCCTCCCTGTCT and 5′-CAGCCTCAGTGTCTGTGAGC, respectively. Forward primers for FR1 of Vλ1, Vλ2, Vλ3 and Vλ4 families are: 5′-GGTGACTCAGCCTCCCTCT, 5′-TGTCCATGTCTCTGGGAGAA, 5′-GATTCCCTCCATGTCTGTGG and 5′-CCTCCTTGGGAACCACAGTA, respectively. Reverse primers complementary to first exons of Cμ, Cγ, Cα and Cε are: 5′-CAGCACTTTGGTTTTGGTAGG, 5′-TTGCAGGTATATGACTGAGAGGAC, 5′-TCACCAGTTCTAGAGTCACAGAGG and 5′-CAGATGTGGGATCATAAGTAGCTG, respectively. The reverse primers that complementary to Cκ and Cλ are: 5′-TGGTTGGAAGATGAAGGCAG and 5′-ACCATAGGCCATGACCATGG, respectively. PCR conditions for all Ig primer combinations were: denaturation at 94°C for 1 min, followed by 34 cycles of denaturation at 94°C for 30 sec, annealing at 62°C for 4 min, and a final single extension period of 68°C for 5 min. All novel Ig sequences generated have been uploaded to GenBank under accession numbers JQ271825 through JQ272176.

The germ-line opossum VH, DH, JH, Vκ, Jκ Vλ, and Jλ gene sequences are in the Somatic Diversification Analyses (SoDA) database (https://dulci.org/soda/) [29]. Ig H and L transcript sequences were analyzed using the SoDA website to determine which germ-line gene segments were contained in each clone and to analyze their junctional diversity and identify P and N nucleotides. Previously published adult opossum Igκ and Igλ transcripts were also included in the analysis presented [23], [25].

### Other Opossum B cell Specific Gene Expression

Opossum CD79a and CD79b genes have been described previously [30]. Primers were designed for the exons flanking introns in each gene for use in RT-PCR. For CD79a they were 5′-CCTGTGAACACGACGGGGGC and 5′-CCGGGACGACAGCGCAGAAA; for CD79b they were 5′-GCTCCTTCAGCGTGACCTGCC and 5′-CTGGGTGCTCGCCCACTGAC. Opossum VpreB3 (GenBank Accession No. JN863116) was recently identified and primers were designed for exons 1 and 2 (5′-AGGAGGGCCTTCTCAGGA and 5-GCTCCTGCTCCTCTTCATTG, respectively) and used in RT-PCR.

## Results

### Initiation of B cell Development in the Opossum

**Figure 1:**
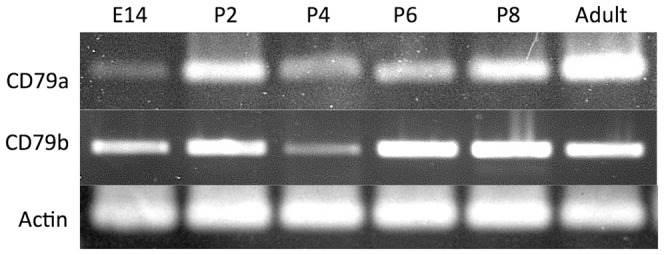
Detection of CD79a and CD79b transcripts in pre- and post-natal opossums. RT-PCR was performed on total RNA from embryonic (E) day 14 and postnatal (P) days 2, 4, 6, and 8, and splenic RNA from an adult opossum as a control. β-actin was included as a positive control.

Previous analyses revealed that productively rearranged and transcribed αβTCR could be detected by RT-PCR within the first 24 postnatal hours in opossums [31]. The same time window was chosen to begin investigating the initiation of B cell development. Two markers of early mammalian pro-B cells are the CD79a and CD79b signal transduction molecules, also known as the Ig-α and Ig-β components of the cell surface B cell receptor (BCR) [32], [33]. The opossum CD79a and CD79b homologues have been characterized previously [30]. Transcription of both CD79a and CD79b was detected by RT-PCR as early as gestational day 14 (E14) (**Fig. 1**). E14 represents the last 24 gestational hours, just prior to birth in the opossum [18].

**Figure 2:**
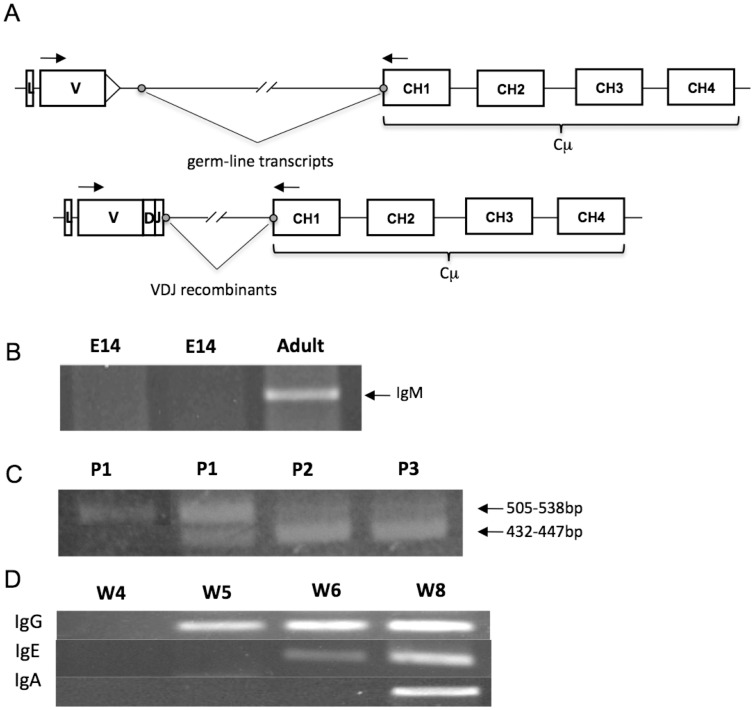
Ig heavy chain gene rearrangement during postnatal opossum development. **A**, RT-PCR with VH1 primer paired with the IgM C region primer using RNA from two independent E14 embryos. Adult spleen RNA is used as a positive control. **B**, RT-PCR products generated using primers specific for VH1 and IgM C region from germ-line, sterile IgM transcripts (505–538 bp range) IgH and complete VDJ recombinants (432–447 bp range) from representative P1, P2, and P3 individuals. **C**, Diagram showing different mRNA splice variants from the germ-line, sterile IgM transcripts (top) and complete VDJ recombinants (bottom). The arrows indicate the relative locations of primers used in the RT-PCR showon in **A** and **B**. The gray circles indicate RNA splice sites. The triangle on the 3′ side of the germ-line V gene indicates the RSS. **D**, Detection of transcritpts from the different IgH isotypes by RT-PCR using VH1 family primer paired with primers specific for the CH1 exon of IgG, IgE, and IgA. Splenic RNA from the animals of the age indicated were used.

Next, to investigate transcription of rearranged IgH chain genes, RT-PCR was performed using primers specific for each of the three opossum VH families paired with a primer for the first exon of each of the opossum IgH chain constant (C) regions (**Fig. 2A** **)**. Tissues from all gestational day 14 (E14) embryos were negative for IgH transcripts (**Fig. 2B**). Only RT-PCR using primers specific for the VH1 family paired with the IgM C region successfully amplified products from animals in the first week of life (**Fig. 2C** **)**. B cells that had switched to IgG, IgE, and IgA were not detected in the first four postnatal weeks in any tissue (**Fig. 2D** and data not shown). Five of 9 postnatal day 1 (P1) opossums tested, which were taken from six different litters, were positive for a PCR product that ranged from 432 to 447 bp in length and, when sequenced, contained productive VDJ recombinants (**Fig. 2C**). All 9 P1 animals tested yielded a PCR product 505 to 538 bp in length that, when cloned and sequenced, was found to encode an un-rearranged germline VH gene and IgM C genes separated by a short fragment of non-coding intervening sequence (**Fig. 2A and C**).

The germ-line VH transcripts detected contained un-recombined VH genes with their recombination signal sequence (RSS) intact and a short stretch of intervening non-coding sequence spliced to the start of the IgM C exons using a cryptic mRNA splice site downstream of the RSS (**Fig. 2C**). The RSS is the sequence recognized by the Recombination Activating Gene (RAG) recombinase complex. These sterile, germ-line VH transcripts were detected in all nine P1 animals and some P2 and P3 animals and likely represent the sterile VH transcripts generated during the initiation of V to DJ recombination in pro-B cells in other species (**Fig. 2B**) [34]. Of 28 independent germ-line transcripts characterized, 26 used the VH1.1 gene segment, which is the most DH proximal of the VH genes in the opossum [21]. The remaining two used VH1.3 and 1.4, respectively, which are the next most DH proximal, functional VH genes [21]. The majority (26 out of 28) germ-line transcripts were spliced to the CH1 exon of the functional IgM locus. The remaining two were spliced to an IgM pseudogene found 5′ of the functional copy in the opossum [21]. Transcripts from productively rearranged H chain VDJ genes were detected in only five of the nine P1 animals tested (**Figs. 2B** **and** **3** and data not shown). All were using VH1 family members recombined to different DH and JH genes and spliced to the functional IgM C region.

**Figure 3:**
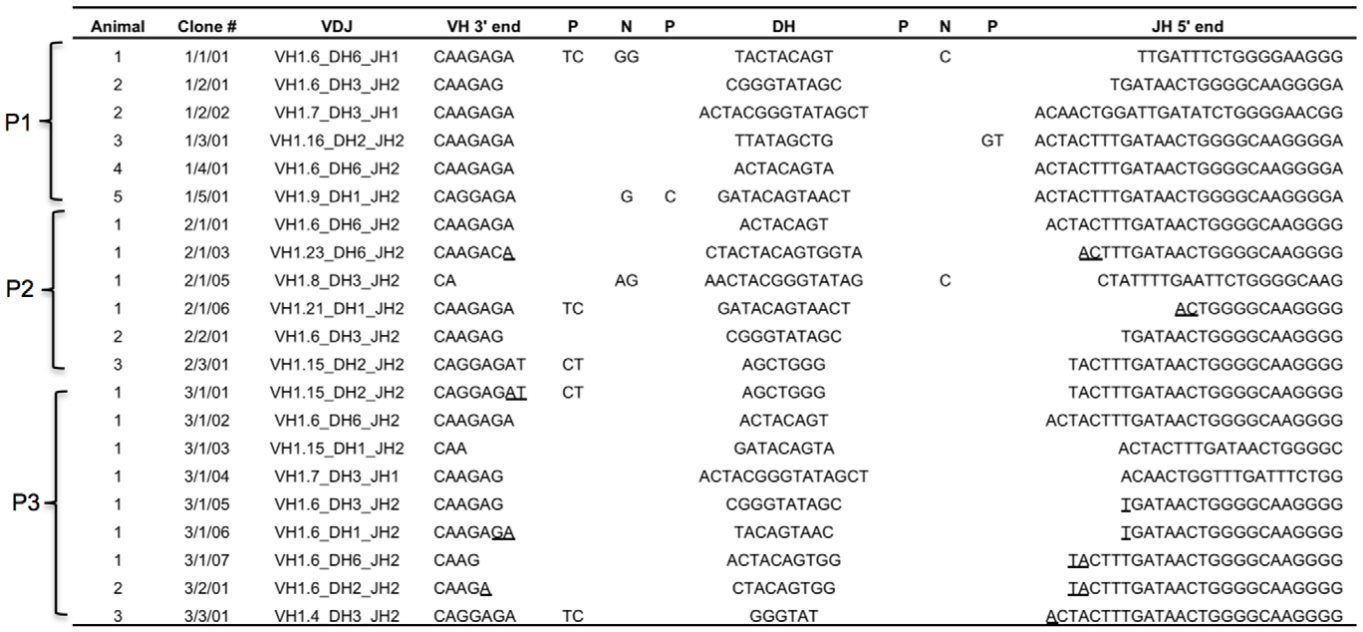
Representative IgH VDJ junctions isolated from P1, P2, and P3 animals. Only the 3′ end of the V through the 5′ end of the J is shown. The age of the animals is indicated on the left and individual opossums in each age group are numbered in the first column, followed by the clone number in the second column. Identification of nucleotides as P, N or D was determined using the SoDA web-based analysis tool. The specific V, D, and J germ-line gene segments used in each clone are indicated in the third column. Nucleotides that represent potential micro-homology at the VD and DJ junctions are underlined.

**Figure 4:**
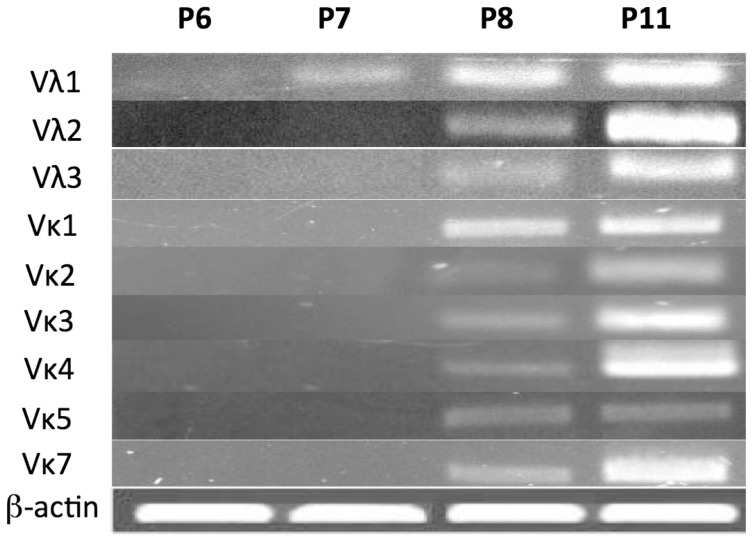
Ig light chain gene rearrangement during postnatal opossum ontogeny. The expression of opossum Ig light chain was determined by RT-PCR at the ages indicated using primers specific for each V family and the relevant C gene. β-actin was included as a positive control.

Marsupial Ig light chain V gene segments, Vκ and Vλ, are more abundant and diverse in the genome than are the heavy chain V genes [21], [23], [25], [27]. To amplify Igλ transcripts, primers specific for FR1 of each Vλ family members were used with a primer that could pair with all eight known Cλ genes. The same approach was applied to Igκ transcripts where there is a single known Cκ. The earliest time-points where Igλ and Igκ transcripts containing VJ rearrangements could be detected were P7 and P8, respectively (**Fig. 4**).

**Figure 5:**
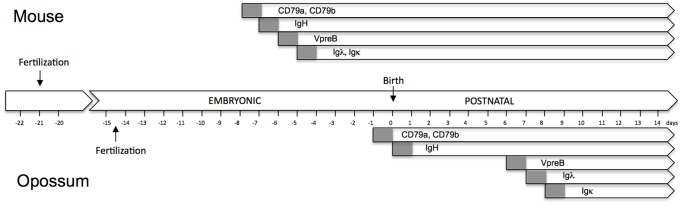
Diagram comparing key points in B cell development in mice and opossums relative to fertilization and birth. The periods of gene expression are indicated by horizontal bars. The gray zone indicates a 24 hour period over which expression is initiated.

In summary to this point, B cell lineage commitment has occurred by the last 24 hours of gestation with full-length IgH chain gene transcription beginning in the first 24 hours postpartum. This is the earliest time-point for evidence of committed B cells so far described for a marsupial. However, full B cell maturation is delayed, with Ig light chain expression being detected seven days after the initiation of IgH expression (**Fig. 5**). The light chain expression immediately follows the transcription of the *VpreB3* gene in postnatal opossum, which occurs on postnatal day 6 at the earliest [35]. *VpreB3* is the only known surrogate light chain in marsupials [35].

### IgH Repertoire Development in the Opossum

The diversity of expressed IgH chain rearrangements during opossum ontogeny was also investigated. Transcribed VDJ rearrangements were amplified by RT-PCR from animals at different ages and the products were cloned and sequenced. For each P1 animal, eight to twelve clones from at least two independent PCR reactions were analyzed. Productive H chain VDJ rearrangements were successfully amplified from only five of the nine P1 animals (**Fig. 3**). In three P1 animals only a single, productive rearrangement was detected and the clones were different and unique to each individual. Of the remaining two animals, one yielded two different productive rearrangements, and the other one productive and one non-productive rearrangement (P1 animals no. 2 and 5 in **Fig. 3**, and data not shown). In the case of the non-productive rearrangement, it is missing most of CDR3 and FR4 having rearranged the VH1.23 gene to approximately the last third of JH1 (not shown). Whether this was due to excessive trimming during VDJ recombination or direct VH to JH recombination is not obvious and there is no evidence of a cryptic RSS heptamer in JH1 (not shown). Of the six different productive VDJ rearrangements, three used the VH1.6 gene. The remaining three used VH1.7, VH1.9, and VH1.16 gene segments, respectively (**Fig. 3**). Four different DH segments were used in these rearrangements (DH1, 2, 3, and 6) with two (DH3 and DH6) each being used twice. There are only two JH used in the opossum; two of the six P1 clones used JH1 and the four remaining used JH2. Animals at ages P2 and P3 were also examined and found to be similar to P1 individuals. Four out of six of the P2 and P3 age animals, like P1 animals, yielded only a single VDJ recombinant. The other two animals, one P2 and one P3 in age, yielded much greater diversity of sequences with four unique VDJ from the P2 and seven unique VDJ from the P3 (**Fig. 3**).

Of the 21 unique VDJ rearrangements isolated from P1, P2, and P3 animals, only three contained N-additions (**Fig. 3**). Previous analyses of fetal mouse IgH rearrangements revealed the absence of N-additions was associated with the use of micro-homology to direct VDJ recombination [13]. Evidence of micro-homology, however, was observed in less than half (eight of 21) of the VDJ recombinants isolated from P1, P2 and P3 animals (**Fig. 3**). Although N-additions were rare in the first three postnatal days, they were not completely absent. This is consistent with previously published evidence that transcription of TdT can be detected in the first 24 postnatal hours in newborn opossums [31].

In the first three postnatal days, nine different VH genes were used; however there was some bias in that nearly half (10 of 21) used VH1.6. This bias, however, did not appear to correlate with evidence of micro-homology at the V(D)J junctions (**Fig. 3**). There was also no apparent positional bias for the VH being used in the early developing repertoire as the nine VH used were scattered throughout the locus [21]. Furthermore, the RSS are nearly identical between VH1 genes and were also not likely to influence recombination frequency (not shown).

**Figure 6:**
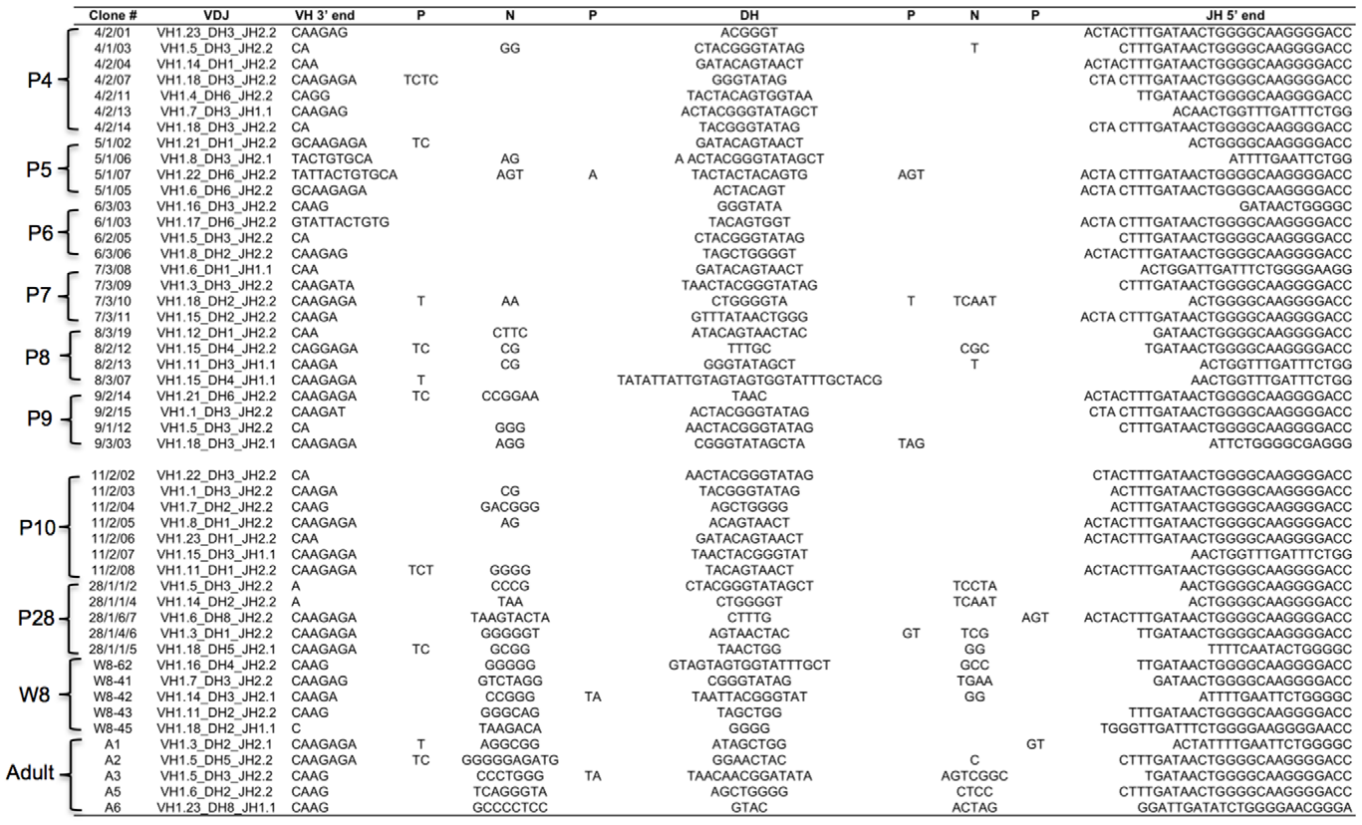
Representative IgH VDJ junctions, showing only the end of the VH through the start of the JH for P4 through adult animals. The age of the animals is indicated on the left and individual opossums in each age group are numbered in the first column, followed by the clone number in the second column. Nucleotides identified as P, N or DH, as determined using the SoDA website are indicated at the top. The specific V, D, and J germ-line gene segments used in each clone are indicated in the third column. Putative micro-homology at the VD and DJ junctions is underlined.

There are 19 functional VH1 family members in the opossum *IgH* locus [21]. By P8, 18 of them were used in expressed VDJ rearrangements. The exception is VH1.13 that, although appearing functional in the germ-line, was never found at any age (**Figs. 3** **and** **6**). The frequency of clones containing N-additions ranged from 21 to 37% for P4 through P8. By P11 the frequency of CDR3 containing N-additions jumped to 45%. By week 8 the frequency was 100%, where it remained into adulthood (**Fig. 6**). The frequency of clones containing N-additions paralleled the number of N-nucleotides added to the junctions to some degree. For the first six days the average number of N-additions remained less than one. In P7 to P11 animals the length of the CDR3 contributed by N-additions increased to an average greater than two. By P28 the average number of N-additions increased to 4.1. By wk 8 and beyond the average number of N-additions was substantially larger at 8 to 11 nucleotides added per CDR3. Similarly the CDR3 length remained a fairly similar 29 to 33 nucleotides from P1 through P11 and then there is an increase after P11 to greater than 35 that are associated with increased N-additions (**Fig. S1**).

The opossum *IgH* locus contains nine DH genes and all nine are functional and can be found in the expressed repertoire (**Fig. S2**). Four different DH gene segments (DH1, 2, 3, and 6), however, account for 85% all the VDJ recombinants isolated. These also happen to be the four shortest germ-line DH genes [21]. All four were found in VDJ recombinants in the P1 repertoire and remained the dominant DH genes at early time-points (**Fig. 3**). Eight of the nine DH, including the four used most frequently have ORF in two of three frames, but this did not appear to influence their use (not shown). The only bias for DH gene segments used is a preference for shorter DH segments that remains throughout adulthood. The contribution that the DH genes made to CDR3 length did not vary as the opossums mature (range of 9 to 14 nucleotides). Rather the increase in CDR3 length in older animals was due to increased N-additions as described above. The opossum *IgH* locus contains six JH segments, of which four appear functional based on genomic sequence [21]. However only two, JH1 and JH2, are used and these are the two immediately upstream of the functional IgM C region (**Figs. 3** **and** **6**) [21]. They were not used equally, however, as JH2 was found in 85% of all VDJ rearrangements and this bias begins at P1 (**Fig. 3**).

### Opossum L Chain Diversity in Early Ontogeny

**Figure 7:**
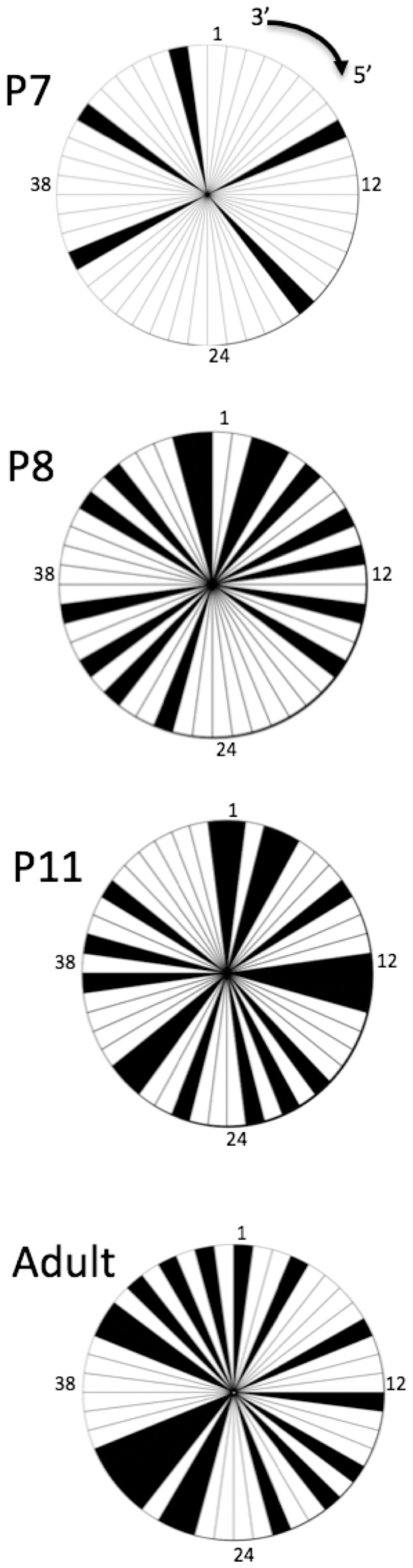
Expression of Vλ1 genes in opossums at different ages. Pie diagrams representing the detection of transcripts containing Vλ1 gene segments in opossums at ages of P7, P8, P11 and Adult. Vλ1 gene segments are ordered clockwise on the pie diagrams from 3′ to 5′ in the Igλ locus. Due to the large number of Vλ1 gene segments, not all are labeled in the figure. For positional reference, four gene segments are numbered on four positions of the compass. Black filled pie wedges indicate positive detection of that specific V gene in a Igλ transcript by RT-PCR at the specific ages.

There are 64 total V genes in the opossum Igλ locus and based on nucleotide identity they have been grouped into four subfamilies [21], [23]. Of the 64 V genes, 58 appear functional based on genomic analysis [21]. The Vλ4 family contains only a single gene segment that appears functional, however this gene was never detected in expressed VJ rearrangements at any age (not shown). As shown previously, Igλ transcripts were first detected at P7 but only by RT-PCR using Vλ1 specific primers (**Fig. 4**). Vλ1 is the largest V family, containing 83% (48 out of 58) of the functional Vλ genes. The diversity of Vλ-Jλ rearrangements at P7 was limited and only a total of six different recombinants were isolated from two different individuals. These six Vλ1 clones used five different Vλ genes, which were scattered across the 1.5 megabase region of the opossum Igλ locus (**Fig. 7**) [21]. Indeed, there was no evidence for positional bias for Vλ at any age in the opossum (**Fig. 7**) As in other mammalian species, the opossum Igλ locus contains tandem J-C pairs; there are eight opossum Cλ each with their own upstream Jλ [21], [23]. Early on there was a bias with 5 of the 6 P7 rearrangements detected using Jλ8-Cλ8, which is the most Vλ proximal of the Jλ-Cλ pairs (**Fig. S3**). By P8, Igλ transcripts containing V genes from all three expressed Vλ families could be detected (**Fig. 4**). Of 38 Igλ transcripts sequenced from P8, almost half (16 out of 38) also used the Jλ8-Cλ8 gene cluster. This bias for the most V proximal Jλ-Cλ pair, however, appeared to be gone by P11 (**Fig. S3**).

**Figure 8:**
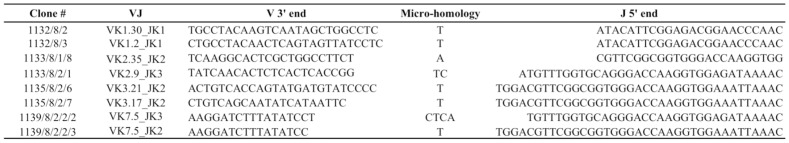
Representative partial Igκ transcripts collected from P8 opossums. The clone numbers are indicated in the first column. The specific Vκ and Jκ germline gene segments used in each clone are indicated in the second column. Nucleotides that could have come from either the germline Vκ or Jκ (*i.e.* evidence of micro-homology at the VJ junctions) are in the fourth column.

Igλ transcripts were also collected from animals at ages P9, P11, and week 8 for comparison. Expressed Igλ sequences from adult opossum were also available from a previous study and were included in this analysis [23]. A comprehensive analysis revealed the CDR3 length remained unchanged over developmental time, however the CDR3 of Vλ1 recombinants were 25% longer than Vλ2 and Vλ3 due to Vλ1 gene segment coding regions being longer (not shown). Approximately a third to a half of all Vλ CDR3 contained evidence of micro-homology in the P7 through P11 animals (not shown, however see **Fig. 8** for similar results with Igκ, described in the next paragraph). By week 8 there was no evidence of using micro-homology. Less than half of the L chain clones in the opossum contained N-additions at any age, consistent with down-regulation of TdT later in pre-B cells undergoing L chain rearrangement as has been seen in other species [36].

Functional Igκ V-J rearrangements were first detected at P8 (**Fig. 4**). There are 122 V genes divided into seven V families in the opossum Igκ locus, however only 82 Vκ appear functional based on genomic sequence [21]. The opossum Igκ locus contains a single Cκ gene and previously only two Jκ had been identified [21], [25]. While analyzing expressed Vκ-Jκ recombinants, a third Jκ designated Jκ3, was found and mapped 3′ of Jκ1 and Jκ2 in the Igκ locus (**Fig. S4**). Using Jκ3 to search the opossum whole genome sequence a fourth J, Jκ4, was also found. V genes from six of the seven Vκ families could be detected in the expressed Igκ repertoire at P8 (**Fig. 4**). Missing was the Vκ6 family, which contains only a single V gene and was not found in any Igκ transcript (not shown). As with Vλ, there was no positional bias in the selection of Vκ genes used at P8; Vκ genes from across the locus were found to be used on the first day Igκ transcripts could be detected (not shown) [21]. As with Igλ clones, the Igκ CDR3 lengths did not vary significantly with age, and most clones lacked N region additions (not shown). Jκ1, 2 and 3 were used starting on P8 (**Fig. S3**). However, although appearing functional, Jκ4 was never used. The expressed Vκ-Jκ rearrangements were analyzed for evidence of micro-homology at the junctions (**Fig. 8**). Micro-homology could be detected in clones at early time-points independent of which Vκ or Jκ was used suggesting that it does not significantly influence bias in the Vκ-Jκ genes recombined.

In summary, the diversity of expressed Igκ and Igλ light chains is greater in early B cells than is heavy chain diversity. This observation is consistent with a previous hypothesis that marsupials may rely more heavily on light chain germ-line diversity than heavy chain for developing their antibody repertoire [27]. Indeed, developing opossums appear to express a repertoire of Igλ and κ chains at the earliest time-points that is equivalent to that of the adult.

## Discussion

Analyses of the immune responses of opossums and other marsupial species, some dating back nearly half a century, provided evidence that the ability to generate endogenous antibody responses developed during postnatal life, when neonates are being exposed to exogenous antigens [4], [7]–[9]. The results presented in this study are entirely consistent with these earlier observations. The results support B cell ontogeny in the gray short-tailed opossum being initiated perinatally with CD79a and CD79b transcription detectable as early as the last 24 hours of gestation. Initiation of VDJ recombination at the IgH chain locus begins within the first 24 postnatal hours as evident by P1 animals having at least initiated transcription of germ-line, sterile VH transcripts. Although not previously characterized in a marsupial, the sterile VH transcripts are likely the equivalent of those described in mice and humans and associated with chromatin remodeling and initiation of V to DJ recombination [34], [37]. Only half of the P1 animals tested had progressed to having B cells with complete IgH chain VDJ rearrangements, consistent with P1 opossums being on the cusp of pre-B cell development. Transcription of both Rag-1 and TdT in the first postnatal 24 hours was previously demonstrated in newborn opossums, consistent with the results presented here that there are committed lymphocytes undergoing V(D)J recombination at this time point [31]. Following VpreB3 expression and subsequent light chain gene rearrangement, the earliest time-point opossums would appear to have B cells with a mature BCR would be P7 [35].

Our study provides the first evidence for marsupial B cell lineage commitment starting prior to birth. Another recent study of early marsupial B cell development investigated CD79a and CD79b transcription in pouch young of the Australian tammar wallaby (*Macropus eugenii*) [30]. Transcription of these BCR subunits was detected in P10 wallaby bone marrow, cervical thymus, and lung, as well as CD79a alone in spleen, gut and blood tissues. P10 was the earliest time point tested in the wallaby study and no other component of the BCR was investigated. Furthermore, initiation of B cell development in tammar wallabies at time points earlier than P10 had been previously shown by the detection of CD79b^+^ cells by immunohistochemistry in gut tissue of pouch young at P7 [12]. A study of B cell development in the Australian brushtail possum (*Trichosurus vulpecula*) reported the detection of IgM transcripts in pouch young spleen and liver by P10 [38]. This study also did not investigate earlier time-points, nor did it investigate the diversity of IgH chains at early developmental stages. It is noteworthy, however, that *T. vulpecula* B cells that had switched to IgG were not detected until after two months of age. This time point is significant in that it is after the point when pouch young transition from being firmly attached to the teats to suckling intermittently similar to newborn placental mammals [39]. Similarly an IgG switch was not detected in the opossum until the fifth postnatal week, which is also immediately after opossums have ceased to be firmly attached to the teats and start suckling intermittently and moving around independently [18]. *M. domestica* is a pouch-less marsupial and once the young detach from the teats they are maintained in a nest or cling to their mother’s side and back, rather than being held in a pouch. This transition may be associated with increased exposure to environmental antigens after the young are more actively rooting around. Although they are not yet weaned, there is likely an increased likelihood of ingesting pathogens and other antigens, driving B cell maturation into the memory pool and Ig class switch. If antigen exposure is obligatory for driving the early isotype switch in marsupials this would be in contrast to placental species where fetal B cells initiate class switch independently of antigen exposure [40], [41].

One significant difference between the results presented here and the brushtail possum study is the appearance of B cells that had switched to IgA. In *T. vulpecula*, IgA transcripts were detected as early as P18, prior to IgG [38]. In the opossum, B cells that had switched to IgA were detected late, in the eighth postnatal week in both the spleen and a mucosal site, the gut. This may represent species-specific differences, but is more likely due to differences in the history of the animals used. In the experiments described here, the opossums are from a long-term captive colony kept under pathogen-free conditions. In the *T. vulpecula* study the animals were wild-caught and likely at greater risk of prior exposure to pathogens that may have stimulated an early IgA response [38]. If indeed antigen exposure drives isotype-switching in marsupials only after they have detached from the teat this would suggest that the teat is forming a tight barrier against antigen intrusion that, along with other innate immune mechanisms, helps to provide protection to the newborn.

The use of somatic mutation, such as gene conversion, to diversify the primary antibody repertoire is often found in species that lack germ-line VH gene diversity (reviewed in ref. 42). Birds and some placental species such as rabbits are noted for their reliance on somatic mutation for generating a diverse primary Ig repertoire; a process that typically takes place in a gut associated lymphoid tissue such as the *Bursa of Fabricius* or the appendix [42]. In such species both the Ig heavy and light chain loci have limited germ-line V diversity. Mice and humans in contrast have diverse germ-line V repertoires and do not use somatic mutation to further diversify the primary repertoire. Opossums, like rabbits and birds, have limited germ-line VH diversity but show little evidence of modification of the primary repertoire by somatic mutation [21], [22]. Whether or not opossums undergo somatic hypermutation of the kind that leads to affinity maturation in the secondary repertoire remains to be determined.

Opossums, like other marsupials, have diverse light chain V genes. We previously hypothesized that marsupials depend upon this light chain diversity for their overall Ig diversity [27]. Other investigators have hypothesized that germ-line heavy and light chain V genes co-evolve in that species with high or low germ-line diversity in heavy chain V genes have corresponding high or low diversity in light chain V genes [43]. Marsupials appear to break this rule by having low germline VH gene diversity but high light chain V gene diversity [21], [23], [25], [27]. The results presented here remain consistent with this hypothesis, as there is early use of a diverse Vλ and Vκ repertoire in the developing opossum.

Four separable stages of B cell development were distinguished in the opossum as defined by the expression of CD79a and CD79b, IgH chain genes, VpreB3, and Ig light chain genes. In fetal mice the transition from heavy chain rearrangement, VpreB expression, and light chain rearrangement and expression occurs over a one to two day period [44]. This process is surprisingly extended in the newborn opossum, occurring over a period of a nearly a week. The delay appears to be primarily due to the lag time between rearranging the IgH chain genes and expression of VpreB3. The reason for this lag-time is not entirely clear. It is possible that B cells that have successfully rearranged their H chain undergo cell division to take advantage of a successful H chain rearrangement by producing daughter clones that undergo independent light chain rearrangements. Mouse B cells that have rearranged their IgH chain genes have been shown to undergo five or six rounds of cell division prior to light gene rearrangement [45]. This may provide the opportunity to develop multiple independent B cell lineages all taking advantage of successful individual heavy chain VDJ recombination.

There is limited or complete absence of addition of N-nucleotides to the VDJ junctions of IgH genes in opossum neonatal B cells. However, there is no evidence that this results in any particular bias in the selection of VH gene segments early in B cell development. This may be, in part due to limited VH germ-line diversity in this species [21]. In other words, use of one VH gene over another may not significantly alter the repertoire. What would then be the advantage for early opossum IgH chains to lack the contribution of N-additions to CDR3 diversity? It is clear that early developing opossum T cells are using TdT to add N-additions to the junctions of TCRα chain genes at the same time-point in development [31]. One possible explanation is provided by recent observations by Schelonka and colleagues who found that reconstituting mice with B cells from TdT-deficient bone marrow resulted in a more rapid repopulation of the spleen and peritoneal cavity compared to wild-type bone marrow [15]. They hypothesized that the lack of N-additions in early B cells favored clones that easily pass through a developmental checkpoint and therefore dominated the early seeding of lymphoid tissues. This, however, appears to be a biological trade-off. B cells lacking H chain N-additions may populate secondary lymphoid sites more quickly, but at the expense of their ability to respond to a variety of pathogens due to limited diversity [15]. It is possible that marsupials are gambling on this trade-off and using early B cells to rapidly populate tissues and drive the development of the secondary lymphoid organs, while relying on maternal Ig absorbed through the milk for protection. Although opossums do not receive any maternal Ig trans-placentally they do rapidly absorb antibodies from the milk soon after the initiation of suckling [46]. Maternal IgA in milk is transferred early in lactation and is likely responsible for regulating gut bacterial flora and protecting against pathogens in newborn marsupials [47].

Many aspects of early postnatal B cell development in the opossum resemble fetal B cell development in humans and mice. The increase in IgH chain CDR3 length in the opossum during the first postnatal week is similar to the fetal to adult transition reported for mice and humans [48], [49]. Similarly is the general lack of N-additions to the H chain VDJ junctions [13]. There is little evidence, however, for bias in the VH genes used in the earliest opossum H chain rearrangements as has been seen for mice [13], [48]. This may not be surprising since, as pointed out in the previous paragraph, there is not much diversity to select from in the germ-line opossum VH repertoire [21]. By the time the young opossum begins to leave the teat its antibody repertoire is as diverse, or nearly as diverse, as that of an adult animal.

In conclusion, this is the first study to determine the earliest time points for the initiation of B cell development in a marsupial and to investigate the ontogeny of the antibody repertoire. B cell development in the opossum is detectable in late stage pre-natal embryos, however completion of B cell development is wholly postnatal, consistent with earlier studies on the generation of antibody responses in newborn marsupials. These observations are critical for improving marsupials as models of immune system development, function and evolution.

## Supporting Information

Figure S1 **Comparision of CDR3 length, P and N nucleotide addition, and DH length in opossum IgH transcripts during postnatal development.** The analysis is based on 244 unique IgH CDR3.
(TIF)

Figure S2 **DH usage frequency in opossums IgH transcripts.** Analyses of the usage frequency of nine DHs in IgH clones obtained from all ages detected. The length of the coding region of each of the germ-line DH is shown below.
(TIF)

Figure S3 **Frequency of Jλ (top) and Jκ (bottom) use in opossum L chains at different ages.** Column chart representing the precentage of clones containing the indicated J segments genes in opossums at the indicated ages.
(TIF)

Figure S4 **Partial map of the opossum Igκ locus.** The location of two new identified Jκ (Jκ3 and Jκ4) gene segments are indicated in the map of opossum Igκ locus.
(TIF)
