# Supplementary figures and images for: Early Postnatal B Cell Ontogeny and Antibody Repertoire Maturation in the Opossum, Monodelphis domestica
Source: PLoS One. 2012 Sep 24;7(9):e45931. doi: 10.1371/journal.pone.0045931 (PMC3454362; doi:10.1371/journal.pone.0045931)

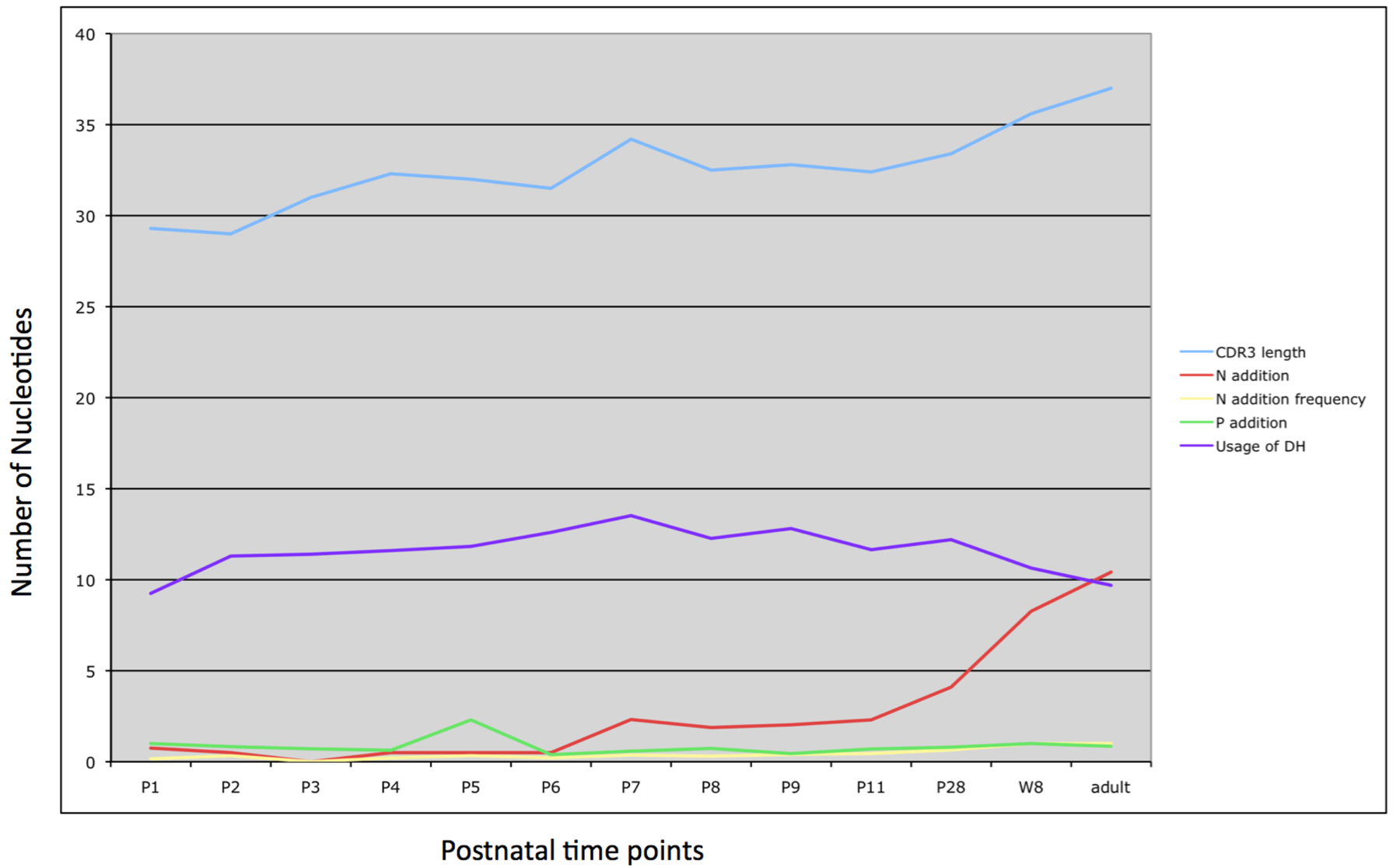

Supplement: Figure S1 — Comparision of CDR3 length, P and N nucleotide addition, and DH length in opossum IgH transcripts during postnatal development. The analysis is based on 244 unique IgH CDR3. (TIF) [file pone.0045931.s001.tif]

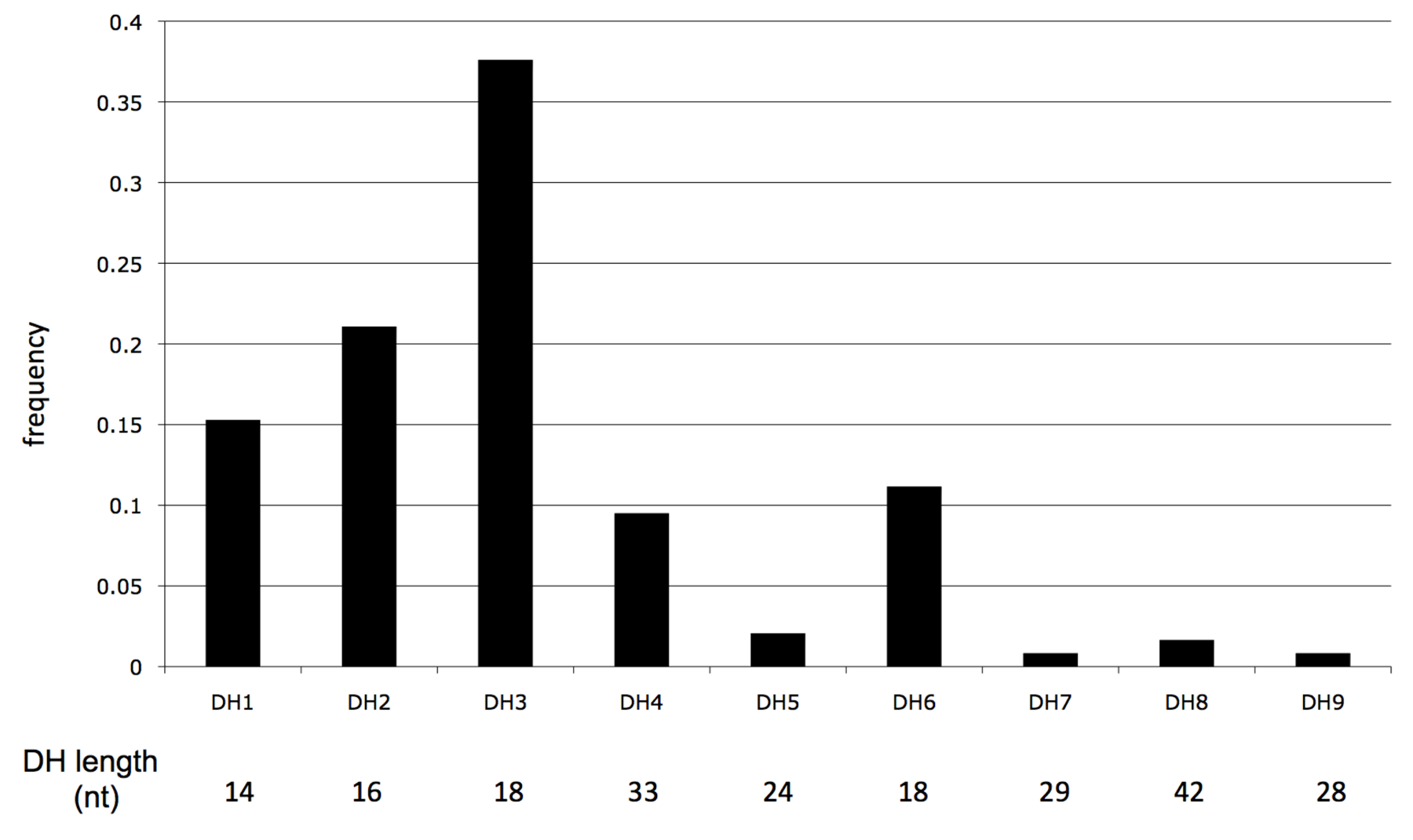

Supplement: Figure S2 — DH usage frequency in opossums IgH transcripts. Analyses of the usage frequency of nine DHs in IgH clones obtained from all ages detected. The length of the coding region of each of the germ-line DH is shown below. (TIF) [file pone.0045931.s002.tif]

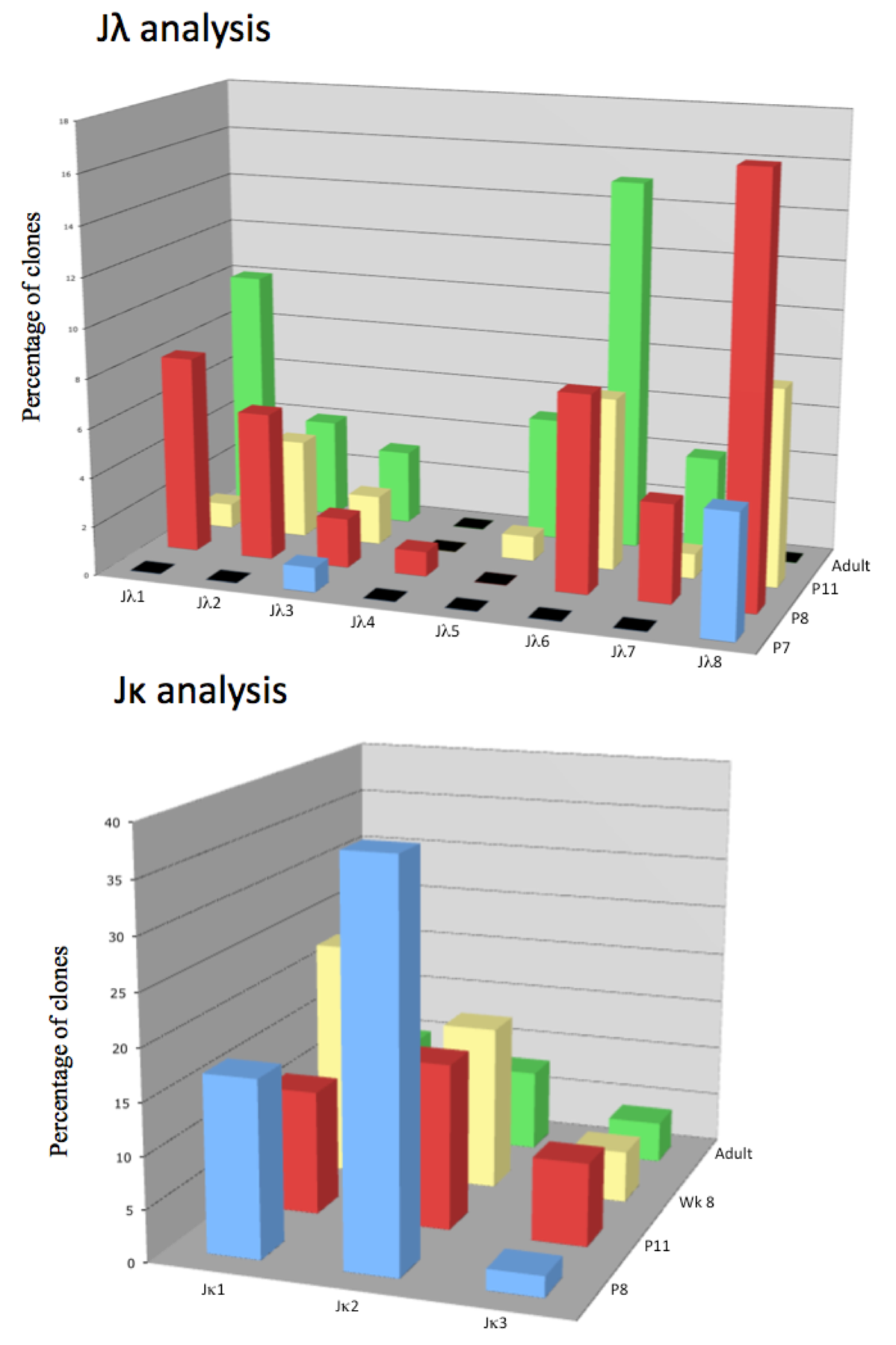

Supplement: Figure S3 — Frequency of Jλ (top) and Jκ (bottom) use in opossum L chains at different ages. Column chart representing the precentage of clones containing the indicated J segments genes in opossums at the indicated ages. (TIF) [file pone.0045931.s003.tif]
